# MR imaging features of benign retroperitoneal extra-adrenal paragangliomas

**DOI:** 10.1038/s41598-017-04753-y

**Published:** 2017-07-03

**Authors:** Yanguang Shen, Yan Zhong, Haiyi Wang, Lu Ma, Yingwei Wang, Jingjing Pan, Kun Zhang, Zhonghua Sun, Huiyi Ye

**Affiliations:** 1 Department of Radiology, Chinese PLA General Hospital, Beijing, China; 2 Department of Radiology, Chinese Navy General Hospital, Beijing, China; 3 Department of Medical Radiation Sciences, Curtin University, Perth, 6102 Australia

## Abstract

The goal of this study was to retrospectively review the magnetic resonance imaging (MRI) features of retroperitoneal extra-adrenal paragangliomas and to evaluate the diagnostic capabilities of MRI. Twenty-four patients with confirmed benign retroperitoneal extra-adrenal paragangliomas who underwent preoperative MRI and surgical resection were enrolled. The patients’ clinical characteristics and MRI features were reviewed by two radiologists. There were no significant differences in the qualitative and quantitative MRI features were determined by the reviewers. High signal intensity in T2-weighted imaging (T2WI) and diffusion-weighted imaging (DWI) was observed in all tumors. In contrast T1-weighted imaging (T1WI) in the arterial phase, 83.33% of the tumors were clearly enhanced. In 87.5% of cases, a persistent enhancement pattern was observed in the venous and delayed phases, and 12.5% of tumors showed a “washout” pattern. The tumor capsule, intratumoral septum and degenerations were visualized in the tumors and may be helpful in the qualitative diagnosis of extra-adrenal paragangliomas in MRI. MRI was useful in locating the position, determining the tumor ranges and visualizing the relationship between the tumors and adjacent structures. The presence of typical clinical symptoms and positivity of biochemical tests are also important factors in making an accurate preoperative diagnosis.

## Introduction

Primary neurogenic tumors, which constitute 10% to 20% of primary retroperitoneal tumors, typically occur at younger ages and are usually benign^1^. Retroperitoneal extra-adrenal paragangliomas account for 1–3% of retroperitoneal tumors, originate in the paraganglionic system and are the most common benign soft-tissue tumors^2, 3^. Extra-adrenal pheochromocytomas are also referred to as ectopic pheochromocytomas or paragangliomas. In the abdominal cavity, extra-adrenal pheochromocytomas occur primarily in the retroperitoneum, the organ of Zuckerkandl, and the urinary bladder^4, 5^.

Although patients diagnosed with retroperitoneal extra-adrenal pheochromocytomas typically present with hypertension, tachycardia, headache and diaphoresis^6^, other tumors may be completely clinically silent and are only detected incidentally in imaging studies; these tumors are referred to as “incidentalomas”. Any physical contact with these tumors can precipitate cardiac arrhythmias and malignant hypertension^7^. This is the most important reason why urologists and radiologists should, now more than ever, understand the imaging appearance of paragangliomas. Because the MR imaging features of most retroperitoneal soft-tissue masses are nonspecific, the prediction of a specific histologic diagnosis remains a radiographic challenge. To our knowledge, a description of the MRI features of retroperitoneal extra-adrenal paragangliomas has been reported for only studies with small sample sizes; therefore, the purpose of our study was to retrospectively analyze the MRI features of benign retroperitoneal extra-adrenal paragangliomas.

## Results

### Demographic data and clinical characteristics

**Figure 1:**
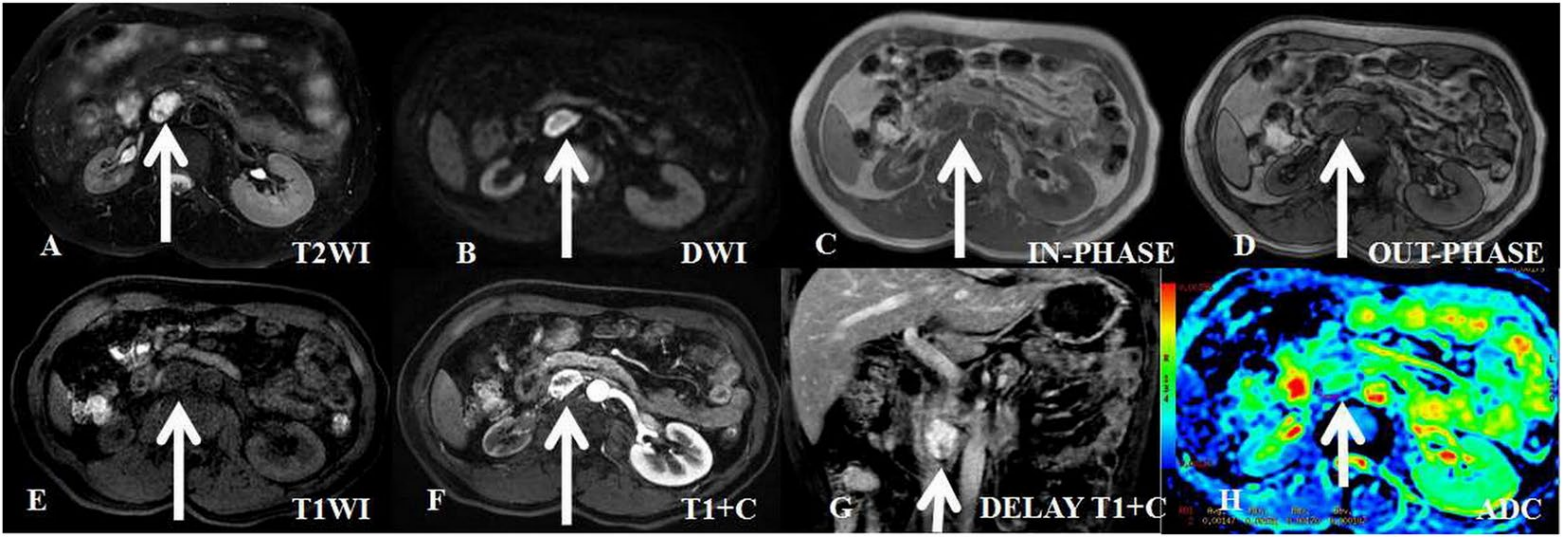
A 49-year-old asymptomatic man with incidentaloma and a histologically proven benign retroperitoneal paraganglioma at the right prevertebral region (between the inferior vena cava and aorta). The transverse and longitudinal diameters of the tumor are 2.61 cm and 3.13 cm, respectively. (**A**) in axial T2-weighted imaging, the tumor demonstrates high signal intensity and isointensity compared with the gluteal muscles. The intratumoral cystic areas exhibit higher signal intensity. (**B**) In axial DWI, the tumor exhibits ring-shaped high signal intensity. (**C**–**E**) T1-weighted imaging (in-phase, out-phase and pre-scanned imaging, respectively); the signal of the tumor is slightly lower than that of the gluteal muscle. (**F**) In contrast T1-weighted imaging during the arterial phase, the tumor is clearly enhanced and non-homogeneous. (**G**) In coronal T1-weighted imaging during the delay phase, the tumor is clearly enhanced and non-homogeneous. (**H**) ADC imaging; the mean ADC value of the ROI of the tumor is 0.00147 mm^2^/s.

**Figure 2:**
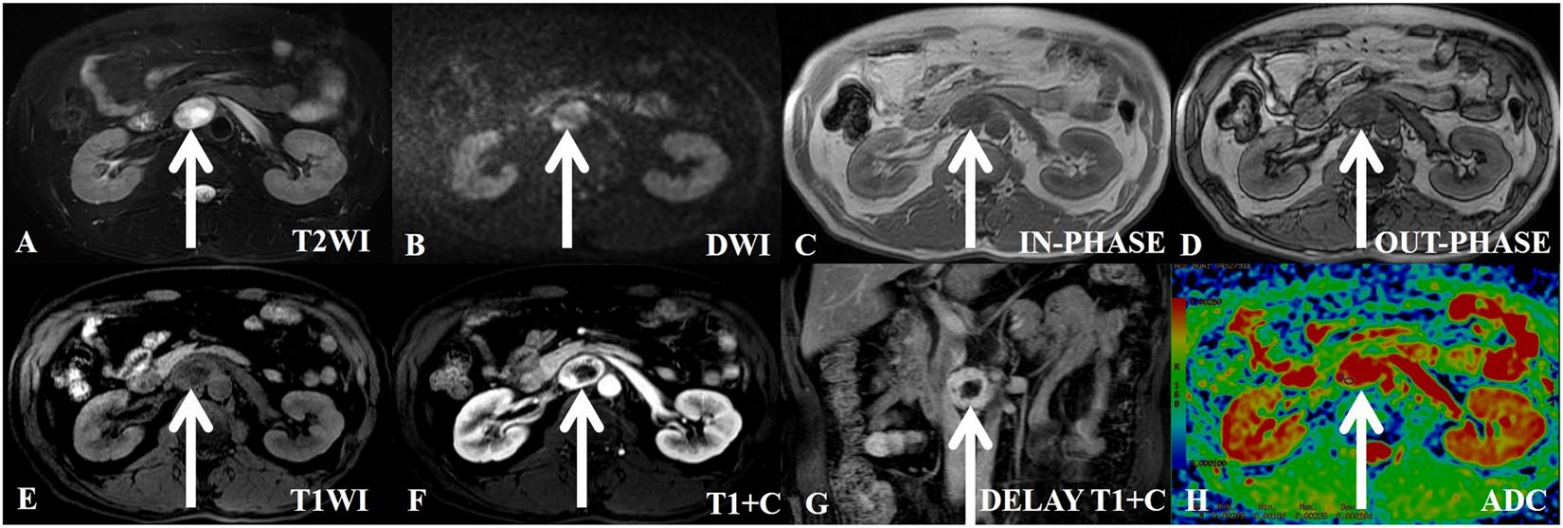
A 47-year-old man with incidentaloma and a histologically proven benign retroperitoneal paraganglioma at the right prevertebral region (among the inferior vena cava, pars horizontalis duodeni and aorta). The transverse and longitudinal diameters of the tumor are 3.92 cm and 3.72 cm, respectively. (**A**) In axial T2-weighted imaging, the tumor demonstrates high signal intensity and isointensity compared with the gluteal muscles. (**B**) In axial DWI, the tumor appears with nodular high signal intensity compared with the spleen. (**C**–**E**) T1-weighted images (in-phase, out-phase and pre-scanned imaging, respectively); the signal of the tumor is slightly lower than that of the gluteal muscle. (**F**) In contrast T1WI during the arterial phase, the tumor is clearly enhanced and non-homogeneously ring-shaped. The center exhibits no enhancement of the necrotic area. (**G**) In coronal T1WI during the delay phase, the tumor shows a continuous and non-homogeneous signal increase. (**H**) ADC imaging; the mean ADC value of the ROI of the tumor is 0.00180 mm^2^/s.

A total of 24 retroperitoneal extra-adrenal paraganglioma lesions in 24 patients were included in this study (Figs 1 and 2). The presenting clinical characteristics of these patients are summarized in Table 1. Ten tumors were found incidentally in 24 patients. Urinary catecholamine (CA) and 24-h urinary vanillylmandelic acid (VMA) concentrations were measured in 22 patients, and 9 tested positive. Eleven patients had hypertension, which was paroxysmal in 3 cases; 3 patients presented with the typical symptoms of pheochromocytoma, including palpitations, headache and diaphoresis. No patient had a history of primary adrenal tumor or other tumors. The mean delay between MRI and surgery was 21 ± 7 days. The tumors were fully excised in all cases, with clear resection margins. The final histologic diagnosis was obtained by laparoscopic surgery (n = 9), robot-assisted laparoscopic surgery (n = 10), or laparotomy (n = 5).

**Table 1:** Patient demographics and clinical characteristics.

Characteristic	Paraganglioma group (n = 24)
No. of patients (female)	24 (12)
*Age (years)	48.04 ± 9.70
Hypertension (n)	11
CA-related symptoms (n)	3
Asymptomatic (n)	10
VMA/24 h (Positive)	22 (9)
CA/24 h (Positive)	22 (9)
^131^I-MIBG positive	9
Other symptoms	
Abdominal mass (n)	2
Lumbago (n)	3
Dysuria (n)	0
Resection of tumor	
Laparoscopic surgery (n)	9
Robot-assisted laparoscopic surgery (n)	10
Laparotomy (n)	5

Note: *Values are mean values ± standard deviations.

Other data are numbers of patients (n).

CA-related symptoms: catecholamine-related symptoms.

VMA: vanillylmandelic acid.

CA: catecholamine.

MIBG: Metaiodobenzylguanidine scintigraphy.

### Imaging features in MRI Scans

The presenting quantitative MR imaging characteristics of mean maximum lesion size, diameter ratio and Apparent Diffusion Coefficient (ADC) values of the 24 patients are summarized in Table 2; there were no significant differences between the assessments made by the two reviewers. Among the 24 lesions, a maximum diameter greater than 5 cm was observed in 17 cases; in the remaining 7 lesions, the maximum diameter was less than 5 cm.

**Table 2:** Quantitative characteristics of retroperitoneal extra-adrenal paragangliomas.

Parameter	Reader 1	Reader 2	*P* value
Tumor maximum size (cm)	5.61 ± 2.11	5.70 ± 2.10	0.875
Diameter ratio (TD/LD)	0.878 ± 0.145	0.855 ± 0.144	0.585
Mean ADC value (×10^−3^ mm^2^/s)	1.541 ± 0.425	1.565 ± 0.425	0.849

Note: Values are mean values ± standard deviations. *P* values were calculated using t tests.

Diameter ratio = Mean TD (transverse diameter)/Mean LD (longitudinal diameter).

ADC: Apparent Diffusion Coefficient.

Findings regarding the qualitative MR imaging features:

1. No statistically significant differences were recorded between the reviewers in lesion location, shape, boundary, microscopic fat, or subacute hemorrhage findings (Table 3). The concordance of the two reviewers for each of the assessed features ranged from good to excellent (from 91.67% to 95.83%) (Table 4).

**Table 3:** Frequency of assessed imaging features in retroperitoneal extra-adrenal paragangliomas.

Imaging Feature	Reader 1	Reader 2	*P* value
**Location**			
Right paravertebral region	8.33 (2/24)	8.33 (2/24)	1.000
Left paravertebral region	37.5 (9/24)	41.67 (10/24)	
Prevertebral region	54.17 (13/24)	50 (12/24)	
Pelvic cavity	0 (0)	0 (0)	
**Shape**			
Round or oval	83.33 (20/24)	87.5 (21/24)	1.000
Irregular	16.67 (4/24)	12.5 (3/24)	
**Boundaries**			
Well-defined	66.67 (16/24)	70.83 (17/24)	1.000
Partly poorly defined	33.33 (8/24)	29.17 (7/24)	
**Microscopic fat**			
YES	12.5 (3/24)	16.67 (4/24)	1.000
NO	87.5 (21/24)	83.33 (20/24)	
**Subacute hemorrhage**			
YES	54.17 (13/24)	50 (12/24)	1.000
NO	45.83 (11/24)	50 (12/24)	
**High signal in DWI**			
Higher	54.17 (13/24)	54.17 (13/24)	1.000
Lower	29.17 (7/24)	25 (6/24)	
Equal	16.67 (4/24)	20.83 (5/24)	
**Necrosis**			
YES	75 (18/24)	70.83 (17/24)	1.000
NO	25 (6/24)	29.17 (7/24)	
**Cysts**			
YES	62.5 (15/24)	66.67 (16/24)	1.000
NO	37.5 (9/24)	33.33 (8/24)	
**Degree of tumor enhancement**			
Avid enhancement	41.67 (10/24)	33.33 (8/24)	0.926
Moderate enhancement	41.67 (10/24)	50 (12/24)	
Slight enhancement	16.67 (4/24)	16.67 (4/24)	

Note: Data are percentages with raw numbers of patients (n) in parenthesis.

*P* values were calculated using χ^2^ tests.

Higher: the high signal of the tumor in DWI is higher than that of the spleen.

Lower: the high signal of the tumor in DWI is lower than that of the spleen.

Equal: the high signal of the tumor in DWI is equivalent to that of the spleen.

**Table 4:** Comparison of the Concordance between Readers for Each Assessed Qualitative Imaging Feature.

Attribute	Concordance
Location	91.67 (22/24)
Shape	95.83 (23/24)
Tumor boundaries	95.83 (23/24)
Microscopic fat	95.83 (23/24)
Subacute hemorrhage	95.83 (23/24)
Necrosis	95.83 (23/24)
Cystic degeneration	95.83 (23/24)
Degree of tumor enhancement	91.67 (22/24)

Note: Values are percentages with raw numbers of patients (n) in parentheses.
2. The results showed that 54.17% of the paragangliomas were located in the prevertebral region, which is close to the aorta and inferior vena cava; no retroperitoneal extra-adrenal paragangliomas were observed in the pelvis.
3. In T1WI, the tumor signal was similar to that of muscle in fat-suppressed T1WI. The signal was heterogeneous in all tumors; areas of less intense signal were observed, pathologically representing necrosis or cysts. Subacute hemorrhage was detected within the tumors of 12 patients (Fig. 3), and microscopic fat was detected in the tumors of 3 patients.

**Figure 3:**
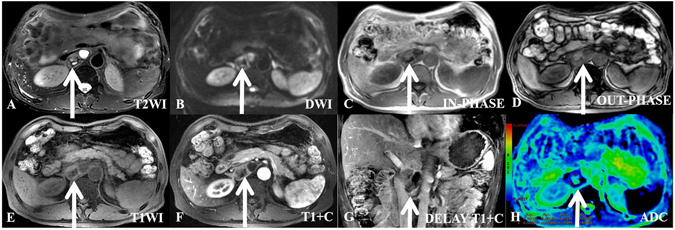
A 60-year-old man with hypertension, positive VMA/24 h and a histologically proven benign retroperitoneal paraganglioma at the right prevertebral region (between the inferior vena cava and aorta). The transverse and longitudinal diameters of the tumor are 2.89 cm and 4.43 cm, respectively. (**A**) In axial T2WI, the tumor exhibits high signal intensity and isointensity compared with the gluteal muscles. The intratumoral septa exhibit low signal intensity. The tumor capsule is shown in T2WI. (**B**) In axial DWI, the tumor appears nodular with ring-shaped high signal intensity. (**C**–**E**) In T1WI (in-phase, out-phase and pre-scanned imaging, respectively), the partial signal of the tumor is higher than that of the gluteal muscle, and most of the signal of the tumor is of slightly lower intensity than that of gluteal muscle. (**F**) In contrast T1WI during the arterial phase, the tumor is clearly enhanced with a non-homogeneous ring. The center exhibits no enhancement of the necrotic area and cysts. (**G**) In coronal T1WI during the delay phase, the tumor exhibits a continuous and non-homogeneous signal increase. The intratumoral septa show a continuous signal increase. (**H**) ADC imaging; the mean ADC value of the ROI of the tumor is 0.00152 mm^2^/s.
4. In T2WI, the tumor signal was much more intense than that of the muscle and liver in fat-suppressed T2WI; in 13 cases, the signal intensity was much higher than that of the spleen; in 6 cases, it was lower; and 5 cases demonstrated equal signals. One paraganglioma demonstrated a fluid-fluid level inside the lesion (Fig. 4).

**Figure 4:**
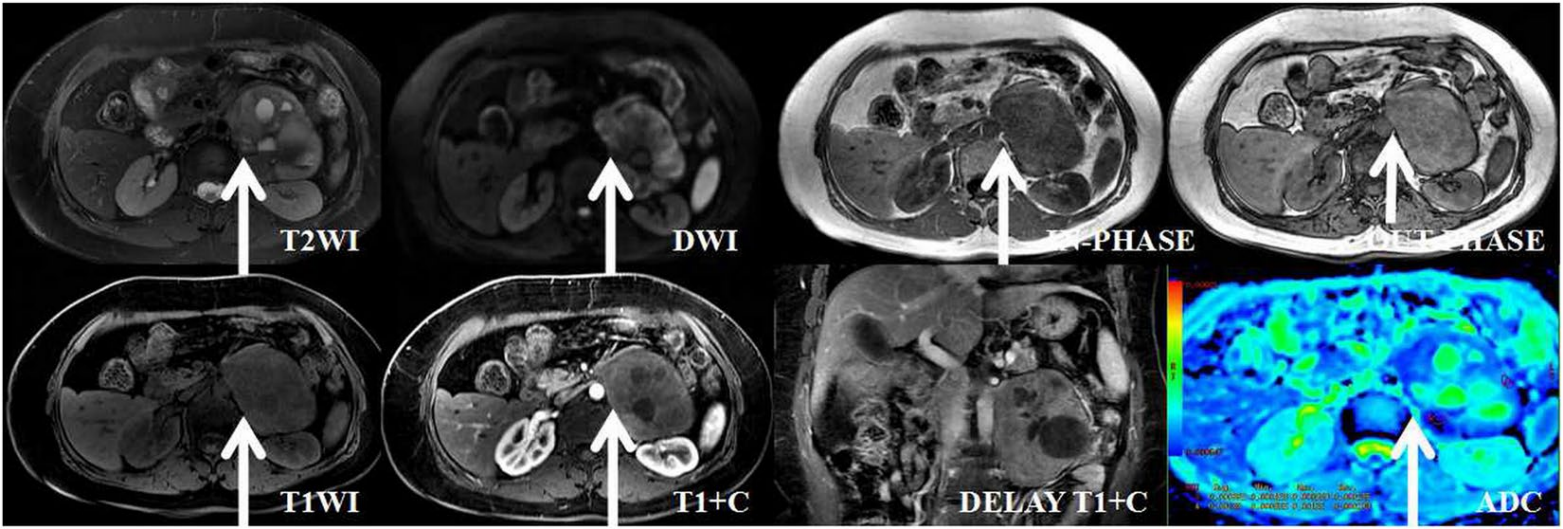
A 53-year-old woman with dizziness and hypertension and a histologically proven benign retroperitoneal paraganglioma at the left paravertebral region (adjacent to the left adrenal gland). The transverse and longitudinal diameters of the tumor are 8.04 cm and 9.71 cm, respectively. (**A**) In axial T2WI, the tumor exhibits mixed signal intensity, including high signal intensity, slightly high signal intensity, and isointensity, compared with the gluteal muscles. The signs of fluid-fluid levels can be seen. The intratumoral septa exhibit low signal intensity. (**B**) In axial DWI, the tumor appears with a ring of high signal intensity. (**C**–**E**) In T1WI (in-phase, out-phase and pre-scanned imaging, respectively), the signal of the tumor is slightly lower than that of the gluteal muscle in in-phase imaging and pre-scanned imaging, and exhibits a linear and sheet-like high signal in out-phase imaging. (**F**) In contrast T1WI during the arterial phase, the tumor exhibits slight and non-homogeneous enhancement. The center exhibits no enhancement of the necrotic area. (**G**) In coronal T1WI during the delay phase, the tumor is slightly enhanced. (**H**) ADC imaging; the mean ADC value of the ROI of the tumor is 0.00130 mm^2^/s.
5. In DWI, the high signal intensity of tumors was heterogeneous; the signal intensity of 13 tumors was much higher than that of the spleen; in 6 cases, it was lower; and in 5 cases, the signal was equal. The shape of the high signal was ring-shaped (n = 11 cases), nodular (n = 5), or a slice (n = 8).
6. More than 80% of the tumors were round or oval, and more than 60% of the tumor boundaries were well-defined. None of the tumors demonstrated infiltrating or encapsulating phenomena. However, the pushing and shifting of nearby structures, such as the pancreas, kidney, inferior vena cava and abdominal aorta, were found for larger tumors.

### MR Imaging features of dynamic enhancement

1. No statistically significant differences were recorded between the reviewers in terms of necrosis, cystic degeneration or degree of tumor enhancement (Table 3).
2. All tumors showed non-homogeneous enhancement after gadolinium administration, with a non-enhancing component showing a fluid signal or necrotic component and peripheral enhancement of the solid elements. Twenty tumors were clearly enhanced in contrast T1WI in the arterial phase. The degree of enhancement of 21 paragangliomas showed a continuous signal increase of mass in the venous and delayed phases (a persistent pattern), whereas only 3 paragangliomas showed “washout” of signal intensity.
3. Cysts and necrosis, which were seen in 24 patients, were divided into three types based on their MR appearance^8^. The first type was characterized by the presence of several small cysts that were separately distributed in the solid tumors (n = 11). The second type was characterized by cysts or/and necrosis located in the centers of tumors (n = 9). The third type was characterized by cysts or necrosis comprising most of the tumor with an irregular wall thickness (n = 5). None of the cysts or necrosis were enhanced in contrast MRI.
4. The intratumoral septa, which were uneven in thickness, were shown in T2WI with low or equal signal intensity and in non-enhanced T1WI with slightly low signal intensity (n = 15); the septa were enhanced in contrast MRI.
5. The tumor capsule was observed in 15 cases in T2WI and in 10 cases in T1WI; the capsule was enhanced in contrast MRI (n = 17).

## Preoperative Diagnostic MRI Findings

The correct preoperative diagnosis of extra-adrenal paraganglioma was made in 75% (18/24) of cases; a diagnosis of incomplete certainty was made in 20.83% (5/24) of cases, and a misdiagnosis was made in one case (4.17%) (Table 5).

**Table 5:** Major clinical data, MR imaging features and diagnostic findings.

Patients	Hypertension	CA-related symptoms	VMA/24 h	CA/24 h	^131^I-MIBG	Location	Degree of tumor enhancement	Preoperative diagnosis of MRI
1	+	−	−	−	+	Left paravertebral region	Slight enhancement	Retroperitoneal extra-adrenal paragangliomas
2	−	−	+	+	+	Prevertebral region	Moderate enhancement	Retroperitoneal extra-adrenal paragangliomas
3	+	−	+	+	+	Prevertebral region	Moderate enhancement	Retroperitoneal extra-adrenal paragangliomas
4	+	−	+	+	+	Prevertebral region	Slight enhancement	Retroperitoneal extra-adrenal paragangliomas
5	+	−	−	−	+	Prevertebral region	Moderate enhancement	Retroperitoneal extra-adrenal paragangliomas
6	+	+	+	+	+	Prevertebral region	Avid enhancement	Retroperitoneal extra-adrenal paragangliomas
7	+	−	+	+	+	Left paravertebral region	Avid enhancement	Retroperitoneal extra-adrenal paragangliomas
8	+	+	+	+	+	Prevertebral region	Moderate enhancement	Retroperitoneal extra-adrenal paragangliomas
9	+	−	−	−	+	Prevertebral region	Avid enhancement	Retroperitoneal extra-adrenal paragangliomas
10	+	+	+	+	−	Prevertebral region	Avid enhancement	Retroperitoneal extra-adrenal paragangliomas
11	−	−	−	−	N.A.	Prevertebral region	Moderate enhancement	Giant lymph node hyperplasia and retroperitoneal extra-adrenal paragangliomas
12	−	−	+	+	−	Left paravertebral region	Slight enhancement	Neurogenic tumor
13	−	−	−	−	−	Left paravertebral region	Slight enhancement	Retroperitoneal extra-adrenal paragangliomas
14	−	−	−	−	N.A.	Right paravertebral region	Moderate enhancement	Neurogenic tumor
15	−	−	−	−	−	Prevertebral region	Avid enhancement	Retroperitoneal extra-adrenal paragangliomas
16	+	−	+	+	−	Prevertebral region	Avid enhancement	Retroperitoneal extra-adrenal paragangliomas
17	−	−	−	−	N.A.	Left paravertebral region	Moderate enhancement	Retroperitoneal extra-adrenal paragangliomas
18	−	−	−	−	N.A.	Prevertebral region	Avid enhancement	Stromal tumor and retroperitoneal extra-adrenal paragangliomas
19	−	−	−	−	N.A.	Left paravertebral region	Moderate enhancement	Retroperitoneal extra-adrenal paragangliomas
20	−	−	−	−	N.A.	Left paravertebral region	Avid enhancement	Retroperitoneal extra-adrenal paragangliomas
21	−	−	−	−	N.A.	Right paravertebral region	Avid enhancement	Retroperitoneal extra-adrenal paragangliomas
22	−	−	−	−	N.A.	Prevertebral region	Moderate enhancement	Neurogenic tumor
23	−	−	−	−	N.A.	Left paravertebral region	Moderate enhancement	Solid pseudopapillary tumor
24	−	−	−	−	N.A.	Left paravertebral region	Avid enhancement	Retroperitoneal extra-adrenal paragangliomas

Note: −, negative; +, positive; N.A., not applicable.

## Discussion

Retroperitoneal extra-adrenal paragangliomas confined to the retroperitoneum are frequently encountered in clinical practice; however, the clinical presentation is often misleading or even absent, and thus diagnostic difficulties are often encountered^9^. To our knowledge, our study includes the largest series to date describing the MRI features of benign retroperitoneal extra-adrenal paragangliomas with DWI and dynamic contrast-enhanced MRI (DCE-MRI).

Many imaging features associated with paragangliomas were observed in the MRI examination. In our study, more than 50% of extra-adrenal paragangliomas were situated in the prevertebral region close to the inferior vena cava and aorta following the aorta-sympathetic chain, consistent with results from previous reports^4, 5, 10^.

Paragangliomas have been described as masses with characteristically high signal intensity or a light bulb-bright signal in T2WI with fat suppression^11^. However, some studies have proposed that this feature is neither specific nor sensitive and may lead to the misdiagnosis of paragangliomas in up to 35% of cases^4, 12^. The high signal intensity is generally thought to be related to the extensive vascular supply of tumors and tumor cells^13, 14^; Dam *et al*.^15^ have proposed that the high signal intensity may be related to tumor catecholamine secretion. Furthermore, we observed that 13 cases had obviously higher signal intensity than that of the spleen in T2WI. The DWI of 13 cases represents the first report of cases in which the high signal intensity is much higher than that of the spleen, and the mean ADC values of all tumors were greater than those of neck paragangliomas^16, 17^. DWI assessment may, therefore, be of significant value for differential diagnosis.

Cyst degeneration, necrosis, subacute hemorrhage and calcifications are common in retroperitoneal extra-adrenal paragangliomas; all these findings tend to be observed in retroperitoneal tumors^18^. Necrotic change was observed in more than 70% of the tumors in our study and tended to occur as paragangliomas increased in size^19^. Brennan *et al*. reported hemorrhagic areas in paragangliomas^20^. Microscopic fat has not been reported in the literature and was not considered a specific feature. Calcifications can occur in all types of neurogenic tumors, without obvious specificity^21^.

DCE-MRI has also been used for tumor detection and characterization and is helpful in distinguishing retroperitoneal extra-adrenal paragangliomas from other retroperitoneal tumors. In our study, 80% of retroperitoneal extra-adrenal paragangliomas exhibited a strong initial signal increase during the arterial phase, consistent with results from other studies^22, 23^. The enhancement features of retroperitoneal paragangliomas in the venous and delayed phases have not previously been reported. Necrotic changes and cysts led to poor central enhancement but exhibited avid peripheral enhancement. Using DCE-MRI, tumor septa of different thicknesses (with low or equal signal intensity in 66.67% of cases in T2WI) demonstrated strong enhancement. Furthermore, we found that MRI was able to distinguish complete from incomplete capsules, thus providing useful information indicating that the tumor has a clear margin that separates it from the surrounding structures. However, it was unclear whether the tumor septa and capsule were characteristics of extra-adrenal paragangliomas in the retroperitoneum; further studies are necessary to confirm that these imaging features are useful for the diagnosis of paragangliomas.

Before surgery, the diagnosis was incorrect in one case (4.17%) on the basis of the MRI examination; a diagnosis with incomplete certainty was made in 20.83% of cases. The major factors for this uncertainty included ignoring less-common locations of extra-adrenal paragangliomas, unfamiliarity with different MR tumor appearances, and the absence of typical clinical symptoms and signs.

Our study has several limitations. First, this was a retrospective study with a relatively small sample size for benign retroperitoneal extra-adrenal paragangliomas, thus reflecting the low incidence of this tumor type. The imaging features observed in our patients were similar to those described in other radiological series, and the small number of cases reflects the rarity of this tumor type. Second, the images reviewed were obtained using different MR instruments using two different field strengths; however, the same imaging protocols were used. Although we demonstrated that field strength had no effect on the ADC measurement of renal tumors between 1.5 T and 3.0 T, we did not include many retroperitoneal tumors; however, this lack should not have significantly affected the imaging features studied. Furthermore, the ADC of various types of retroperitoneal lesions should be compared between 1.5 T and 3.0 T. Third, the lesions presented with predominantly cystic changes, hemorrhage and necrosis, which may have affected the ADC value or signal intensity measurement. Finally, the study included no malignant paragangliomas. We did not conduct a further study to differentiate benign from malignant retroperitoneal paraganglioma tumors.

In conclusion, the high signal intensity in T2WI and DWI, DCE-MRI features, the capsule, the intratumoral septum and degeneration may be helpful in the diagnosis of retroperitoneal extra-adrenal paragangliomas based on MRI. Typical clinical symptoms and positive biochemical tests are also very important in making accurate preoperative diagnoses. Qualitative and quantitative analyses of MRI features play an important role in accurately locating and diagnosing these tumors.

## Materials and Methods

### Patients

This retrospective study was approved by the PLA General Hospital review board, and informed consent was waived due to its retrospective nature. All methods were performed in accordance with the approved guidelines. Pathology and radiology databases were searched to identify all cases of histologically proven retroperitoneal extra-adrenal paragangliomas in which 24 patients had undergone preoperative MRI between July 2008 and February 2016; all patients had undergone surgical resection. The patients’medical histories were reviewed. The search identified 24 patients (12 men and 12 women, mean age: 48.04 ± 9.70 years, age range: 27–67 years) who had a total of 24 benign retroperitoneal extra-adrenal paragangliomas.

### Magnetic Resonance Imaging Protocol

MRI examinations were conducted using a 1.5-T system (n = 2, Signa HDXT, GE Healthcare), a 3.0-T system (n = 16, Signa EXCITE; GE Healthcare) and a 3.0-T system (n = 6, Discovery 750, GE Healthcare). Patients were imaged in the supine position using a surface phased-array coil. Respiratory-triggered transverse and coronal T2-weighted fast spin-echo sequences were initially performed, and this was followed by transverse T1-weighted dual-echo in-phase and out-of-phase sequences and by three-dimensional fat-saturated T1-weighted dynamic contrast-enhanced sequences that were performed during suspended respiration. Gadobenate dimeglumine (MultiHance; Bracco Sine, Shanghai, China) (15 mL) was injected intravenously at a rate of 2 mL/sec using a power injector (Spectris; MedRad, Warrendale, PA), and this was followed by a 20-mL saline flush. DCE-MRI was performed in the transverse plane at baseline (pre-contrast) and during the arterial, venous, and delayed phases. Transverse breath-hold DW images were obtained using a single-shot, spin-echo echo-planar sequence before the administration of contrast material with tri-directional gradients and two b values: 0 and 800 s/mm^2^.

The MR imaging parameters were as follows: T2-w FSE images were acquired using the following parameters: infinite/90–105 ms (repetition time ms/echo time ms); field of view (FOV), 36–44 cm; section thickness, 5 mm; intersection gap, 1 mm; and matrix, 320 × 224. T1-w dual-echo images were acquired using the following parameters: 260/(2.2–2.5; 5.5–5.8) ms; FOV, 36–44 cm; section thickness, 5 mm; intersection gap, 1 mm; and matrix, 256 × 192. For the 3D dynamic contrast-enhanced sequences, the following parameters were used: 3.0–3.9/1.2–1.6 ms; FOV, 34–40 cm; section thickness, 5 mm; interpolated section thickness, 2.5 mm; and matrix, 288 × 224. DWI (5400/50–60 ms; flip angle, 90°; FOV, 36–40 cm; matrix, 128 × 128; section thickness, 5 mm; intersection gap, 1 mm; all directions; one signal acquired) was performed before the DCE-MRI with b values of 0 and 800 s/mm^2^.

## Imaging Analysis

All images were independently reviewed by two radiologists who had 10 and 5 years of experience in the interpretation of abdominal MR images. The two reviewers were blinded to the histologic diagnoses of the lesions at the time of their review. The reviewers evaluated and recorded each lesion for the presence of each of the following features^24, 25^:

*Quantitative MR Image Analysis*:

- Tumor maximum size: the size of each lesion was measured at its single largest diameter in three planes.
- Diameter ratio: two reviewers independently measured the maximum transverse diameter (TD) and the longitudinal diameter (LD) of the mass in coronal section. The means of the maximum TD and LD of the mass in coronal section were recorded. Each measurement was conducted three times, and the mean value was used as the final value to avoid intra- and inter-observer disagreement. The diameter ratio was calculated using the following equation: Diameter ratio = Mean TD/Mean LD.
- ADC values: ADC maps were auto-generated, and region-of-interest (ROI) analysis was performed using an Advantage Workstation (Advantage Workstation, version 4.6, GE Healthcare, Bue, France). ROIs were placed according to the most obvious enhancing region of the retroperitoneal tumors in the arterial phase according to visual assessment, with the aim of avoiding necrosis, hemorrhage and cystic changes (cysts are defined as the part of the lesion showing no enhancement in DCE-MR images). In each patient and for each tumor, three ROIs, each measuring 30–60 mm^2^, were drawn on three target anatomical structures. The mean ADC values in the ROIs on the three targets were calculated for each patient.

*Qualitative MR Image* Analysis (The analysis was based on the following parameters):

1. Position and peripheral location: lesions were determined to be located in the right or left paravertebral region (near the spinal column and psoas muscle, adrenal region or kidney), anterior to the vertebrae (close to the abdominal aorta and inferior vena cava, the origin of the inferior mesenteric artery or peripancreatic location) or in the pelvic cavity.
2. Shape: Tumors were determined to be round/oval or irregular.
3. Margin: Tumors were determined to be well-defined or partly poorly defined.
4. High signal in DWI: The shape of the high signal was divided into nodulars, ring and flaky signal. The signal intensity was compared with that of the spleen in DWI and assessed to be higher, lower, or equivalent to that of the spleen.
5. Microscopic lipid: The area of signal loss in the lesion in out-of-phase T1-weighted images was determined.
6. Subacute hemorrhage: The area of increased T1 signal intensity in unenhanced fat-suppressed T1-weighted images was determined.
7. Cystic degeneration: Areas with signal intensity (SI) equal to that of the cerebrospinal fluid in T2-weighted images, low SI in T1-weighted images, lack of enhancement, and lobulated morphology were assessed.
8. Necrosis: The presence of high SI in T2-weighted images (although not as high as the SI of cerebrospinal fluid), low SI in T1-weighted images, lack of enhancement, and central location within the tumor were assessed.
9. Degree of tumor enhancement: MR imaging was subjectively assessed regarding the degree of mass enhancement compared with that of the renal cortex (avid enhancement, moderate enhancement or slight enhancement) based on Gd-enhanced MR images acquired during the arterial phase.
10. Other MR imaging features were also assessed: intratumoral septa, tumor capsule, fluid-fluid level, subjective assessment of the MR imaging degree and the pattern of mass enhancement during the venous phase and delayed phase.

### Pathologic Diagnosis

All specimens were retrospectively examined by two uropathologists with 10 years of experience in uropathology who were blinded to the MRI findings and who reached consensus.

## Statistical Analysis

Continuous variables were expressed as the means ± SD and were analyzed using independent t-tests for normally distributed data or Mann-Whitney tests for non-normally distributed data. For qualitative variables, Chi square tests were used to compare the sample proportions of the two readers. Kappa coefficients were not used for this determination because the very high prevalence of certain imaging features for many of the binary factors was expected to produce misleadingly low values^24, 26^. All reported *P* values are two-sided and were considered statistically significant at values of less than 0.05. SPSS version 19.0 software was used for all computations.
